# Single-stage matrix-assisted bone marrow-derived cell concentrate implantation for osteochondral talar lesions: a 24-month clinical and MRI follow-up

**DOI:** 10.3389/fsurg.2026.1941433

**Published:** 2026-10-30

**Authors:** Gianfilippo Caggiari, Michelangelo Rinaldi, Alessandro Santandrea, Domenico Mercurio, Giovanni Lugani, Silvia D'Alessandro, Hansheng Deng, Filippo Migliorini, Francesco Pisanu, Carlo Doria, Fabrizio Cortese, Leonardo Puddu

**Affiliations:** 1 Department of Medicine, Surgery and Pharmacy, University of Sassari, Sassari, Italy; 2 Department of Motor, Neurological and Rehabilitation Sciences, Azienda Ospedaliero-Universitaria di Sassari, Sassari, Italy; 3 Department of Orthopaedics and Traumatology, Ospedale Santa Maria del Carmine di Rovereto, Rovereto, Italy; 4 Department of Life Sciences, Health and Health Professions, Link Campus University, Rome, Italy

**Keywords:** bone marrow derived cells, bone marrow stimulation, collagen scaffolds, MOCART, osteochondral lesion of the talus, talus - surgery

## Abstract

**Background:**

The optimal surgical management of larger chronic osteochondral lesions of the talus (OLT) remains debated. This study describes 24-month clinical and MRI outcomes after a combined single-stage matrix-assisted procedure using debridement, bone-marrow stimulation, autologous bone marrow-derived cell concentrate, a type I collagen scaffold, and fibrin glue.

**Methods:**

This exploratory retrospective case series screened consecutive patients treated between January 2016 and December 2020. Twenty-six patients were identified; 15 had sufficiently complete clinical and MRI data for the predefined 24-month complete-case analysis. Eligible lesions were classified as Giannini type II-IIA and Outerbridge grade III-IV. The principal clinical endpoint was the 24-month change in the American Orthopaedic Foot Ankle Society (AOFAS) Ankle-Hindfoot score. Secondary outcomes included Visual Analogue Scale (VAS) pain, 24-month MRI morphology assessed with the MOCART system, and reoperations. Six- and 12-month assessments were part of routine care but were not sufficiently complete for formal longitudinal analysis.

**Results:**

The mean AOFAS score increased from 57.3 ± 12.0 at baseline to 92.4 ± 7.1 at 24 months, an absolute improvement of 35.1 points. The mean VAS score decreased from 4.7 ± 1.2 to 1.8 ± 0.8, an absolute reduction of 2.9 points. At 24 months, complete defect filling was observed in 12 of 15 patients (80.0%), hypertrophic filling in 2 (13.3%), and incomplete filling in 1 (6.7%). Complete integration with adjacent tissue was observed in 14 patients (93.3%); preserved surface morphology and uniform repair-tissue structure were each observed in 12 (80.0%). Mean MOCART score was 83.7 ± 14.4. Three patients (20.0%) underwent arthroscopic reoperation for symptomatic anterior impingement.

**Conclusion:**

In this retrospective cohort, the combined single-stage procedure was associated with improved 24-month pain and functional scores and favorable MRI morphology of repair tissue. The uncontrolled design and multimodal procedure do not establish comparative efficacy, superiority, or an independent cell-specific treatment effect. Larger prospective controlled studies are needed.

## Introduction

Osteochondral lesions of the talus (OLT) involve focal injury of the talar articular cartilage and subchondral bone and are frequently associated with previous ankle trauma (1–4). Symptoms may include pain, swelling, stiffness, mechanical symptoms, and functional limitation. Because articular cartilage has limited intrinsic reparative capacity, persistent lesions can remain symptomatic and may contribute to progressive joint degeneration (5–12).

Treatment is influenced by lesion size, depth, location, stability, symptoms, previous treatment, and associated ankle pathology (13–28). Bone-marrow stimulation techniques such as drilling or microfracture are commonly used for smaller lesions, whereas larger or deeper defects may prompt consideration of osteochondral transplantation, autologous chondrocyte implantation, or scaffold-based regenerative strategies (18–28). Nevertheless, the optimal approach remains controversial, particularly for larger lesions and revision settings.

Interest in bone marrow-derived orthobiologic preparations has increased because they can be harvested, processed, and implanted during a single surgical session (29–34). Importantly, centrifuged bone marrow aspirate is a heterogeneous cellular product and should not be considered equivalent to a purified or culture-expanded mesenchymal stem/stromal cell preparation (34, 35). Transparent description of donor, tissue source, processing, cell characteristics, and delivery site is therefore essential when reporting these procedures (34).

The purpose of the present study was to describe the 24-month clinical and MRI outcomes of a standardized single-stage matrix-assisted procedure for chronic OLT. We specifically evaluated AOFAS and VAS outcomes, MRI morphology of repair tissue using the MOCART system, and postoperative reoperations. Given the retrospective design, small final cohort, and absence of a comparator group, the study was considered exploratory and was not intended to establish the independent efficacy of the cellular component or superiority over other surgical techniques.

## Materials and methods

This was an exploratory retrospective single-center case series of consecutive patients treated between January 2016 and December 2020. The study is reported in accordance with the Strengthening the Reporting of Observational Studies in Epidemiology (STROBE) principles (36). No *a priori* sample-size calculation was performed because all consecutive potentially eligible cases treated during the predefined period were screened.

Patients were eligible if they were 18–50 years old, had persistent symptoms despite at least 6 months of conservative management, had preoperative pain >3 on a 0–10 VAS, and had a chronic OLT classified as Giannini type II-IIA and Outerbridge grade III-IV (37, 38). The operative indication concerned lesions generally larger than 1.5 cm^2^ and less than 10 mm deep within the indication used at the treating center. Exclusion criteria were established ankle osteoarthritis, osteonecrosis, rheumatoid or other inflammatory arthritis, osteochondritis dissecans outside the selected indication, cystic or kissing lesions, clinically relevant lower-limb malalignment, and joint instability requiring a different or additional corrective procedure.

Twenty-six patients were identified. Eleven did not enter the complete 24-month analysis because clinical follow-up was shorter than 24 months and/or postoperative MRI was incomplete. The final analytic cohort therefore consisted of 15 patients (Figure 6). This complete-case approach and the resulting risk of attrition bias were considered in the interpretation of all outcomes.

Formal ethics committee review was not required for this retrospective observational analysis under the institutional and national requirements applicable at the time of the study. The analysis used clinical and imaging data that had already been collected during standard care; no patient underwent randomization, study-specific treatment, additional imaging, or any modification of the therapeutic pathway for research purposes. Data used for analysis were handled with protection of patient confidentiality and were analyzed retrospectively. Written informed consent was available for treatment and use of clinical data for scientific purposes, and written consent for publication was obtained for potentially identifiable images where applicable. Because no formal ethics committee submission or waiver procedure was required for this type of retrospective analysis under the applicable framework, no ethics approval or waiver reference number exists. The study was conducted in accordance with the Declaration of Helsinki and applicable local requirements.

The principal clinical endpoint was the change in the AOFAS Ankle-Hindfoot score (39) from baseline to 24 months. Secondary outcomes were VAS pain (40), 24-month MRI morphology of repair tissue assessed with the MOCART system (41), and postoperative reoperations.

Clinical and imaging assessments at 6 and 12 months were part of routine postoperative care. However, these intermediate assessments were not uniformly archived for all patients in the retrospective dataset. To avoid selective longitudinal reporting, the formal analysis was restricted to baseline and the standardized 24-month endpoint. Intermediate MRI examinations are therefore used only when shown as illustrative examples and not as a cohort-level longitudinal analysis.

Lesion location, size, and depth were determined from the available preoperative MRI and operative documentation and were confirmed during diagnostic arthroscopy. Because the historical records did not consistently document a single standardized planimetric measurement protocol, lesion dimensions are treated as descriptive variables rather than inferential predictors.

All procedures were performed using the same standardized protocol by one senior surgeon assisted by the same surgical team. The patient was positioned supine with the ipsilateral anterior superior iliac crest included in the sterile field. Diagnostic ankle arthroscopy was performed through standard anteromedial and anterolateral portals to assess lesion location, dimensions, depth, cartilage stability, and associated intra-articular pathology. Unstable and nonviable cartilage was debrided to stable margins. Bone-marrow stimulation was then performed with a 9-mm arthroscopic awl, creating multiple microfracture holes approximately 3 mm apart in the exposed subchondral plate (Figure 1).

**Figure 1:**
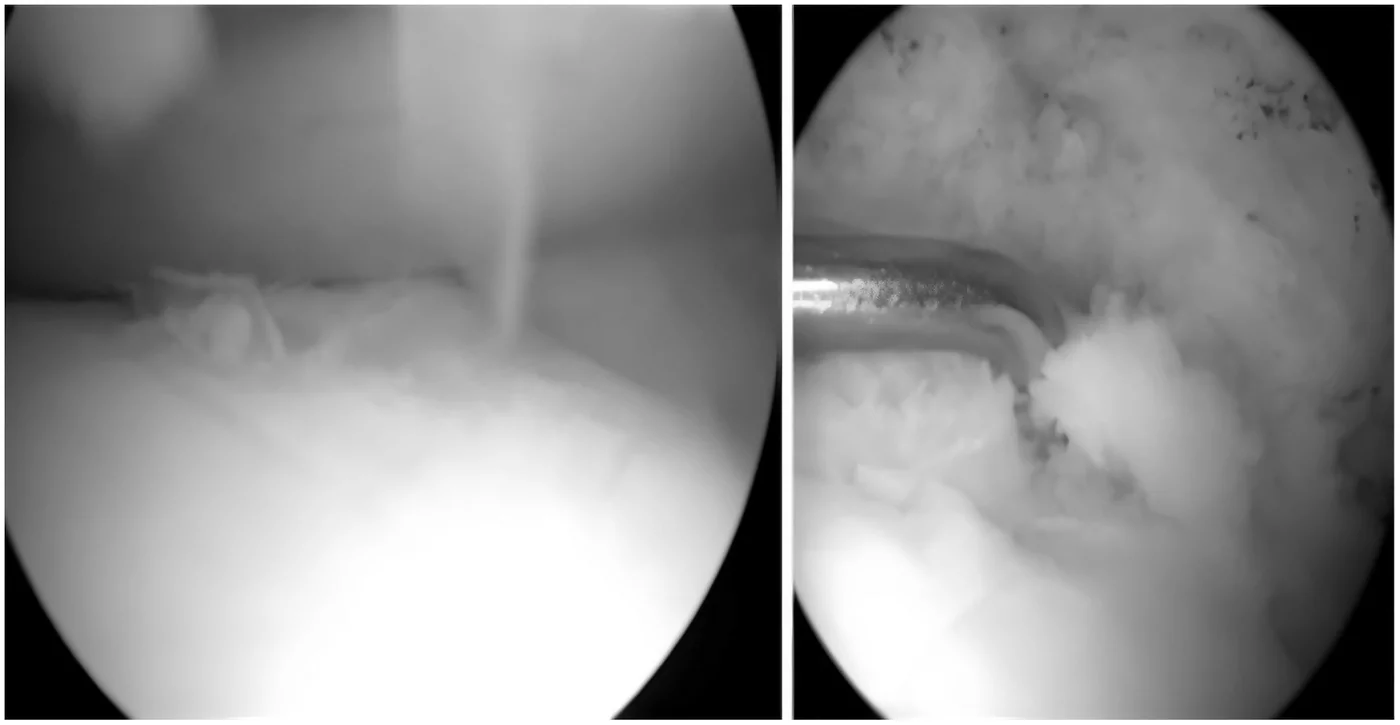
Arthroscopic preparation of the osteochondral lesion and microfracture-based bone-marrow stimulation after debridement to stable margins.

Bone marrow was aspirated from the anterior superior iliac crest under sterile conditions. Approximately 60 mL was collected through multiple aspiration sites using heparinized syringes to limit peripheral-blood dilution. The aspirate was processed intraoperatively using the ResQ™ 60 BMC system (Novagenit, Mezzolombardo, Italy) by centrifugation, yielding approximately 6 mL of concentrated autologous bone marrow-derived cellular product (Figures 2, 3).

**Figure 2:**
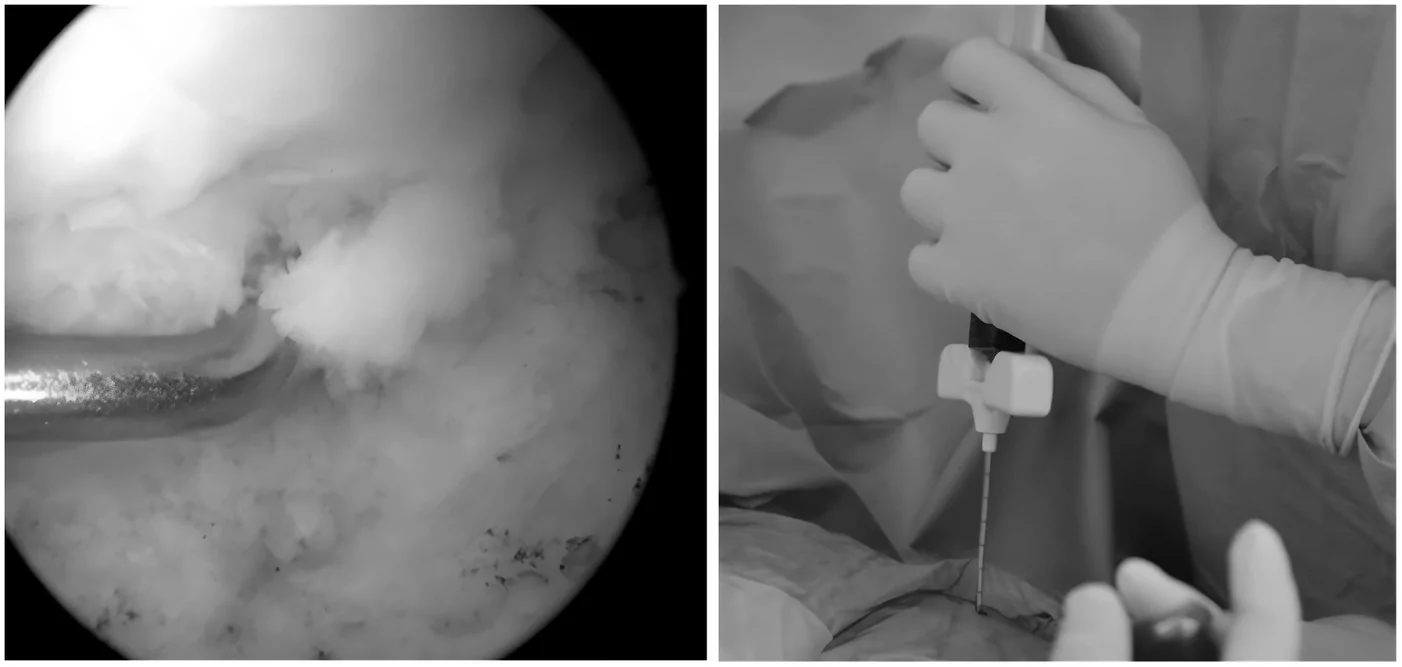
Intraoperative bone marrow harvesting from the anterior superior iliac crest as part of the single-stage procedure.

**Figure 3:**
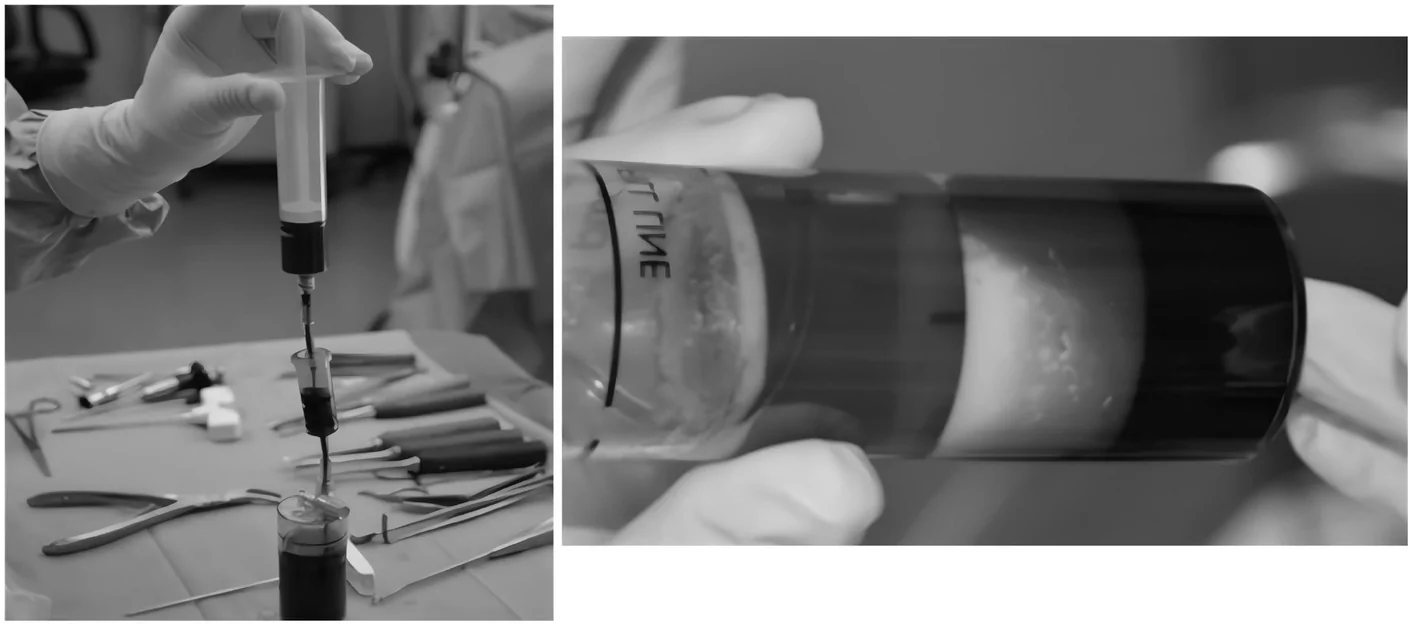
Intraoperative processing and centrifugation of the bone marrow aspirate to obtain the bone marrow-derived cell concentrate.

The implanted product was not an isolated or culture-expanded mesenchymal stem/stromal cell preparation. It represented a heterogeneous, minimally manipulated bone marrow-derived cell concentrate produced by centrifugation. No *ex vivo* expansion was performed, and patient-specific surface-marker characterization or purified MSC quantification was not available in the retrospective dataset. Accordingly, the revised manuscript uses the terms “bone marrow-derived cell concentrate” or “concentrated bone marrow aspirate” when referring to the implanted product, consistent with the reporting principles of the DOSES framework (34) and the limitations of identifying MSCs in fresh bone marrow aspirate concentrate (35).

After removal of nonviable marginal tissue, the lesion bed was prepared under direct visualization through a limited extension of the appropriate arthroscopic portal when required. A highly hydrophilic type I collagen three-dimensional scaffold (IOR-G1, Novagenit, Mezzolombardo, Italy) was tailored to the shape of the chondral defect and loaded with the bone marrow-derived cell concentrate (Figure 4). The scaffold was press-fitted into the lesion and stabilized with fibrin glue. Ankle flexion-extension was performed to confirm adaptation and stability of the implant (Figure 5).

**Figure 4:**
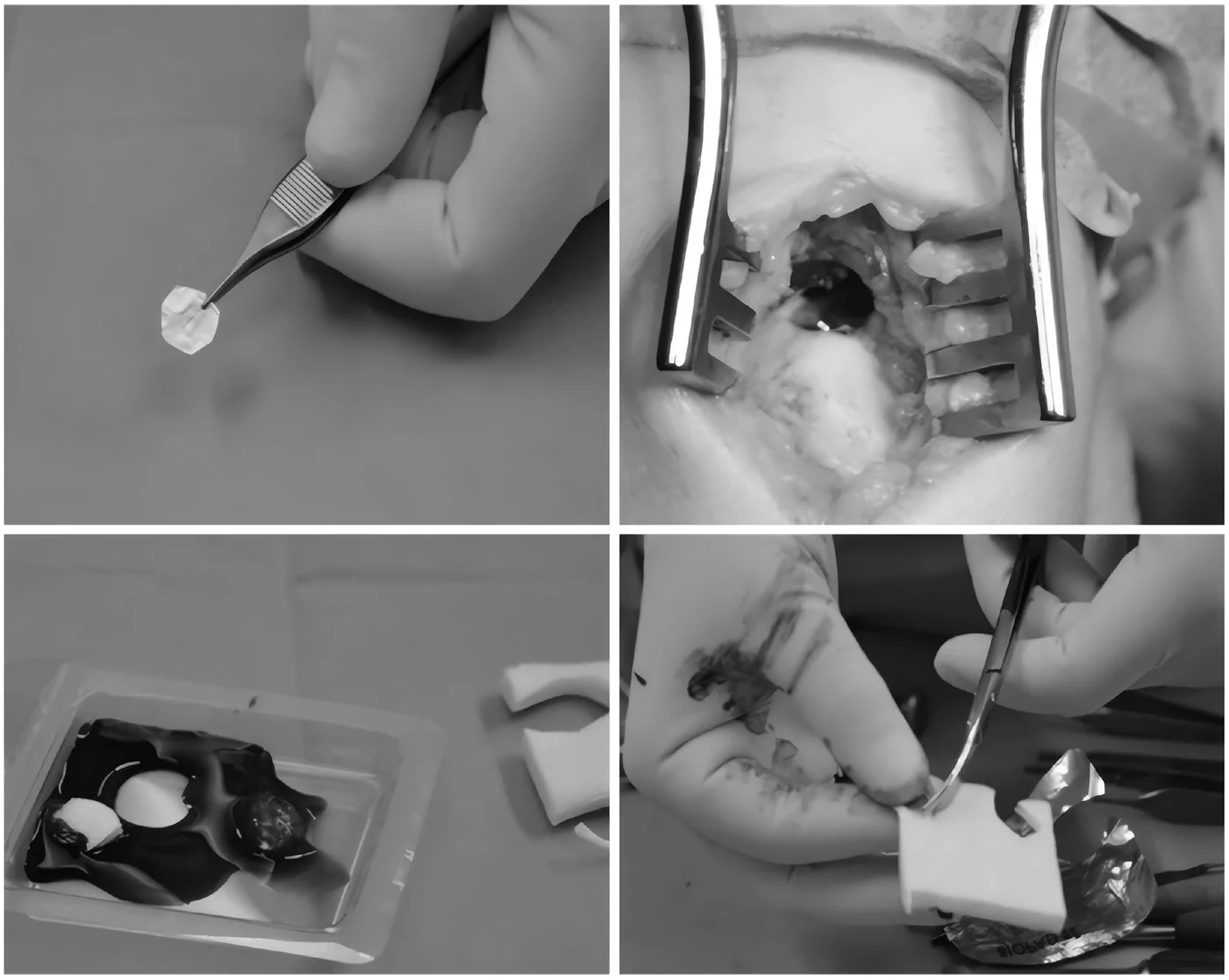
Preparation of the type I collagen scaffold and tailoring to the osteochondral defect before loading with the bone marrow-derived cell concentrate.

**Figure 5:**
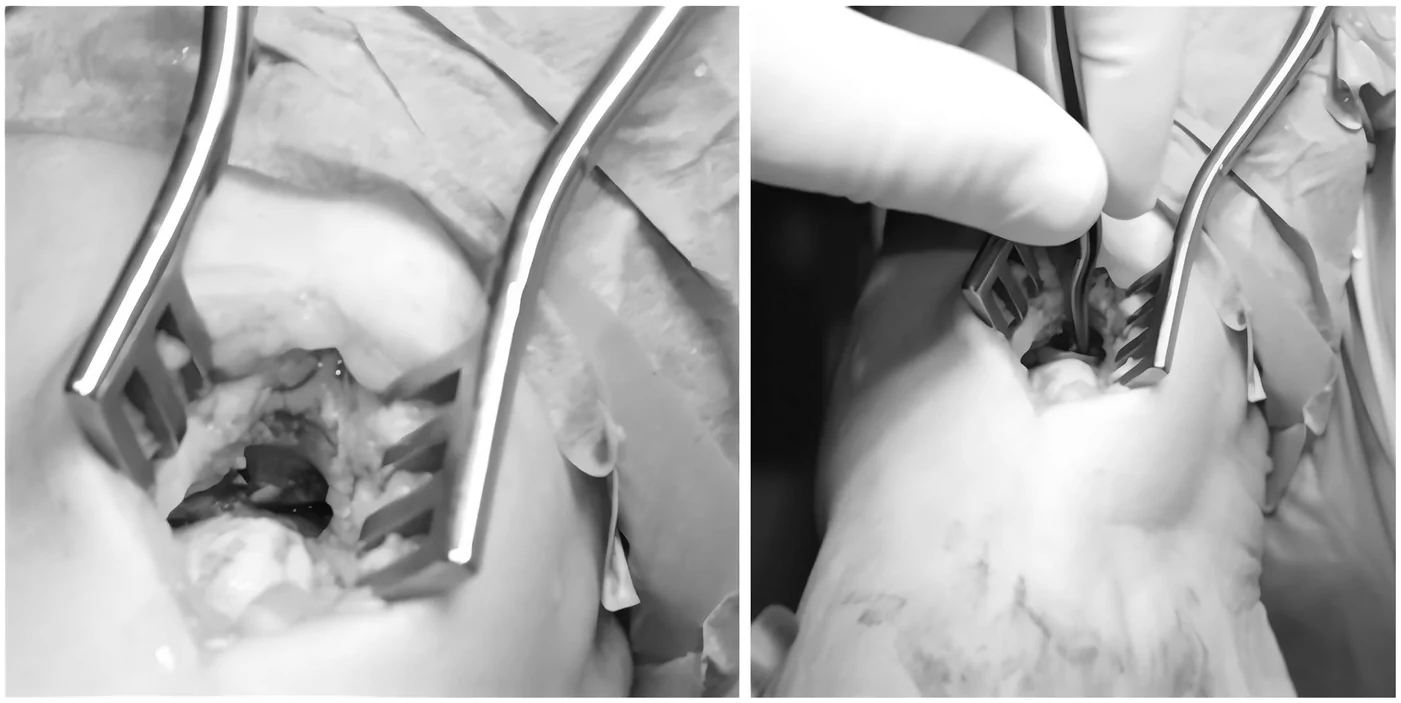
Open visualization of the prepared defect and press-fit implantation/stabilization of the matrix-assisted graft.

Postoperatively, a short-leg splint was used for 2 weeks. After wound review and suture removal, passive and active ankle motion was encouraged. Progressive weight-bearing was permitted from approximately 4 weeks according to symptoms and clinical assessment. Sport and other high-impact activities were restricted for at least 3 months.

Routine MRI included T1- and T2-weighted fast spin-echo sequences and was used to evaluate defect filling, integration with adjacent tissue, surface morphology, repair-tissue structure and signal, subchondral lamina, and other MOCART features (41). Four orthopaedic observers (three senior and one junior) reviewed the MRI examinations. Because the submitted reliability analysis combined several non-equivalent agreement statistics in a very small cohort, formal ICC/kappa/Krippendorff analyses were removed in the revised manuscript. MRI findings are therefore presented descriptively.

MOCART is a morphological imaging score and was not interpreted as evidence that normal hyaline cartilage had regenerated. Throughout the revised manuscript, the MRI findings are described as “repair tissue” or “cartilage-like repair tissue.”

Given the exploratory retrospective design, the small complete-case cohort, the revised statistical analysis is descriptive. Continuous variables are summarized as mean ± standard deviation (SD), with 95% confidence intervals (CIs) for observed means where appropriate. Categorical variables are summarized as counts and percentages. For AOFAS and VAS, the absolute change from baseline to 24 months is reported. No imputation was performed. Formal longitudinal testing, correlations between MOCART and clinical outcomes, and unsupported inferential *P* values were removed. Baseline demographic and lesion summaries in Table 1 refer to the 26-patient screened cohort; clinical and MRI outcomes refer to the 15-patient complete-case cohort.

**Table 1:** Baseline characteristics of the screened cohort (*n* = 26).

Characteristic	Screened cohort (*n* = 26)
Age, years, mean ± SD	34.0 ± 9.5
BMI, kg/m^2^, mean ± SD	25.0 ± 2.5
Sex, *n* (%)	Male, 16 (61.5%); female, 10 (38.5%)
Affected side, *n* (%)	Left, 12 (46.2%); right, 14 (53.8%)
Previous trauma, *n* (%)	Yes, 20 (76.9%); no, 6 (23.1%)
Lesion location, *n* (%)	Medial, 18 (69.2%); lateral, 8 (30.8%)
Time from diagnosis to surgery, months, mean ± SD	16.0 ± 12.4
Lesion area, cm^2^, mean ± SD	1.7 ± 2.1
Lesion depth, mm, mean ± SD	6.0 ± 1.2
Latest follow-up, months, mean ± SD	38.0 ± 3.2

BMI, body mass index; SD, standard deviation. Values are aggregate summaries for all 26 screened patients. The retained files did not contain patient-level baseline data linking the 15 included and 11 excluded patients; therefore, these characteristics must not be interpreted as those of the final 15-patient outcome cohort.

## Results

A total of 26 patients treated during the study period were screened. Eleven patients (42.3%) did not enter the complete 24-month analysis because follow-up was shorter than 24 months and/or postoperative MRI was incomplete, leaving 15 patients in the final analytic cohort (Figure 6). The retained revision files did not identify the 11 excluded cases at patient level; therefore, baseline characteristics could not be recalculated separately for the 15 included and 11 excluded patients.

**Figure 6:**
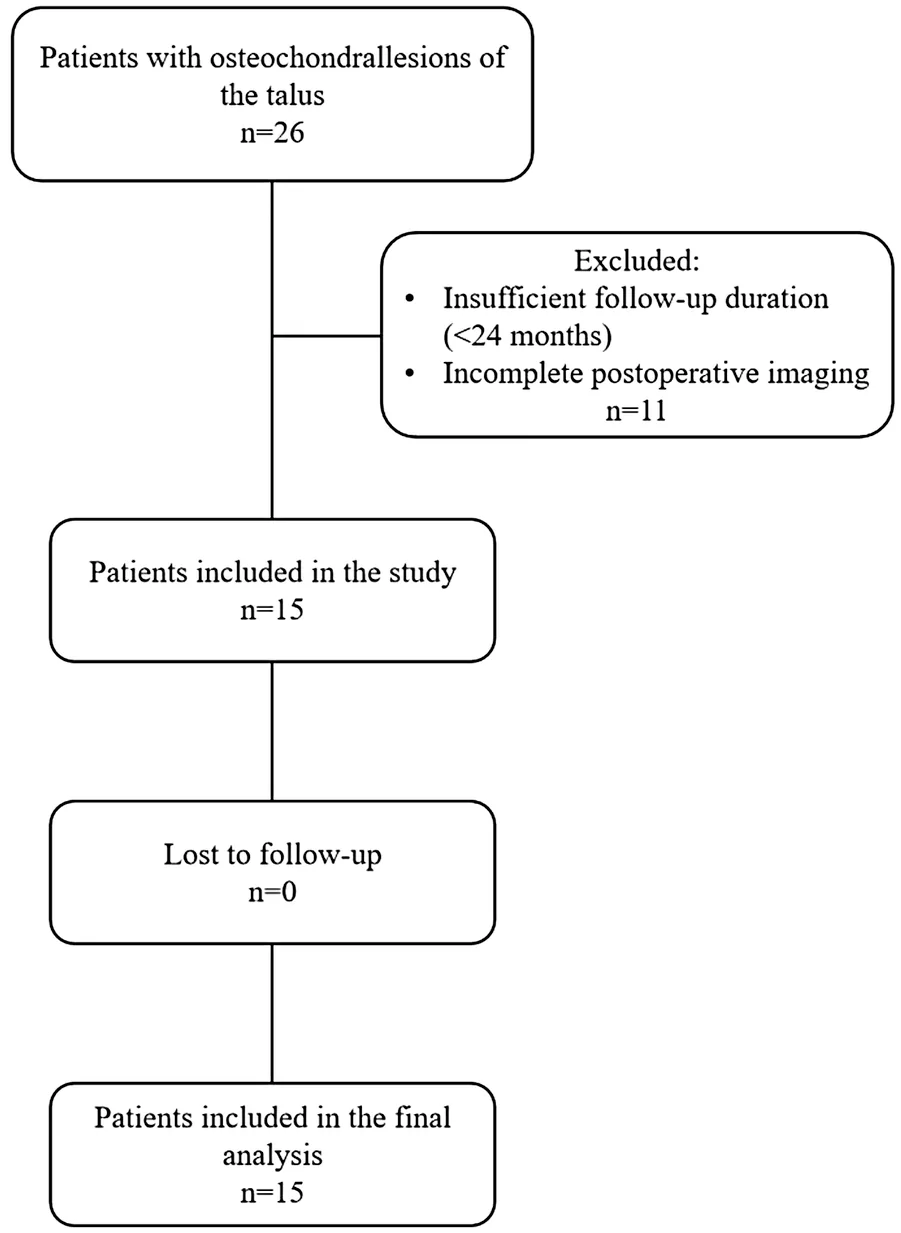
Flow diagram of patient screening and inclusion in the 24-month complete-case analysis.

Aggregate baseline data available for the 26 screened patients showed a mean age of 34.0 ± 9.5 years, mean BMI of 25.0 ± 2.5 kg/m^2^, mean latest follow-up of 38.0 ± 3.2 months, mean interval from diagnosis to surgery of 16.0 ± 12.4 months, mean lesion area of 1.7 ± 2.1 cm^2^, and mean lesion depth of 6.0 ± 1.2 mm. The screened cohort included 16 men (61.5%) and 10 women (38.5%); 12 left ankles (46.2%) and 14 right ankles (53.8%); 20 patients with previous trauma (76.9%) and 6 without (23.1%); and 18 medial (69.2%) and 8 lateral (30.8%) lesions (Table 1). These values describe the screened cohort and are not attributed to the 15-patient outcome cohort.

BMI, body mass index; SD, standard deviation. Values are aggregate summaries for all 26 screened patients. The retained files did not contain patient-level baseline data linking the 15 included and 11 excluded patients; therefore, these characteristics must not be interpreted as those of the final 15-patient outcome cohort.

At 24 months, the mean AOFAS score was 92.4 ± 7.1 compared with 57.3 ± 12.0 at baseline, corresponding to an absolute increase of 35.1 points. The mean VAS pain score decreased from 4.7 ± 1.2 at baseline to 1.8 ± 0.8 at 24 months, corresponding to an absolute reduction of 2.9 points (Table 2). The revised analysis does not describe these changes as statistically significant because the complete paired raw dataset required for a fully documented inferential analysis was not available in the submitted material.

**Table 2:** Clinical outcomes at baseline and 24 months (*n* = 15).

Outcome	Baseline mean ± SD (95% CI)	24-month mean ± SD (95% CI)	Absolute change
AOFAS Ankle-Hindfoot score	57.3 ± 12.0 (50.7 to 63.9)	92.4 ± 7.1 (88.5 to 96.3)	+35.1
VAS pain score	4.7 ± 1.2 (4.0 to 5.4)	1.8 ± 0.8 (1.4 to 2.2)	−2.9

AOFAS, American Orthopaedic Foot & Ankle Society; VAS, Visual Analogue Scale; SD, standard deviation; CI, confidence interval. The reported CIs describe the observed mean at each time point and are not CIs for the paired within-patient change.

At 24 months, MRI demonstrated complete defect filling in 12 patients (80.0%), hypertrophic filling in 2 (13.3%), and incomplete filling in 1 (6.7%). Complete integration with adjacent tissue was observed in 14 patients (93.3%). Preserved surface morphology and uniform repair-tissue structure were each observed in 12 patients (80.0%). The subchondral lamina was preserved in 14 patients (93.3%), while T2 hyperintensity of the repair tissue persisted in 12 (80.0%). The mean MOCART score was 83.7 ± 14.4 (Table 3). A representative serial MRI examination is shown in Figure 7; the 6-month image is illustrative only and is not part of a cohort-level longitudinal analysis.

**Table 3:** Descriptive MRI morphology at 24 months (*n* = 15).

MOCART-related feature	Finding	n (%)
Defect filling	Complete	12 (80.0)
Defect filling	Hypertrophic	2 (13.3)
Defect filling	Incomplete	1 (6.7)
Integration with adjacent tissue	Complete	14 (93.3)
Surface morphology	Preserved	12 (80.0)
Repair-tissue structure	Uniform	12 (80.0)
Subchondral lamina	Preserved	14 (93.3)
Repair-tissue signal	Persistent T2 hyperintensity	12 (80.0)
Overall MOCART score	Mean ± SD	83.7 ± 14.4

MOCART, Magnetic Resonance Observation of Cartilage Repair Tissue. These findings describe repair-tissue morphology and do not provide histological confirmation of normal hyaline-cartilage regeneration.

**Figure 7:**
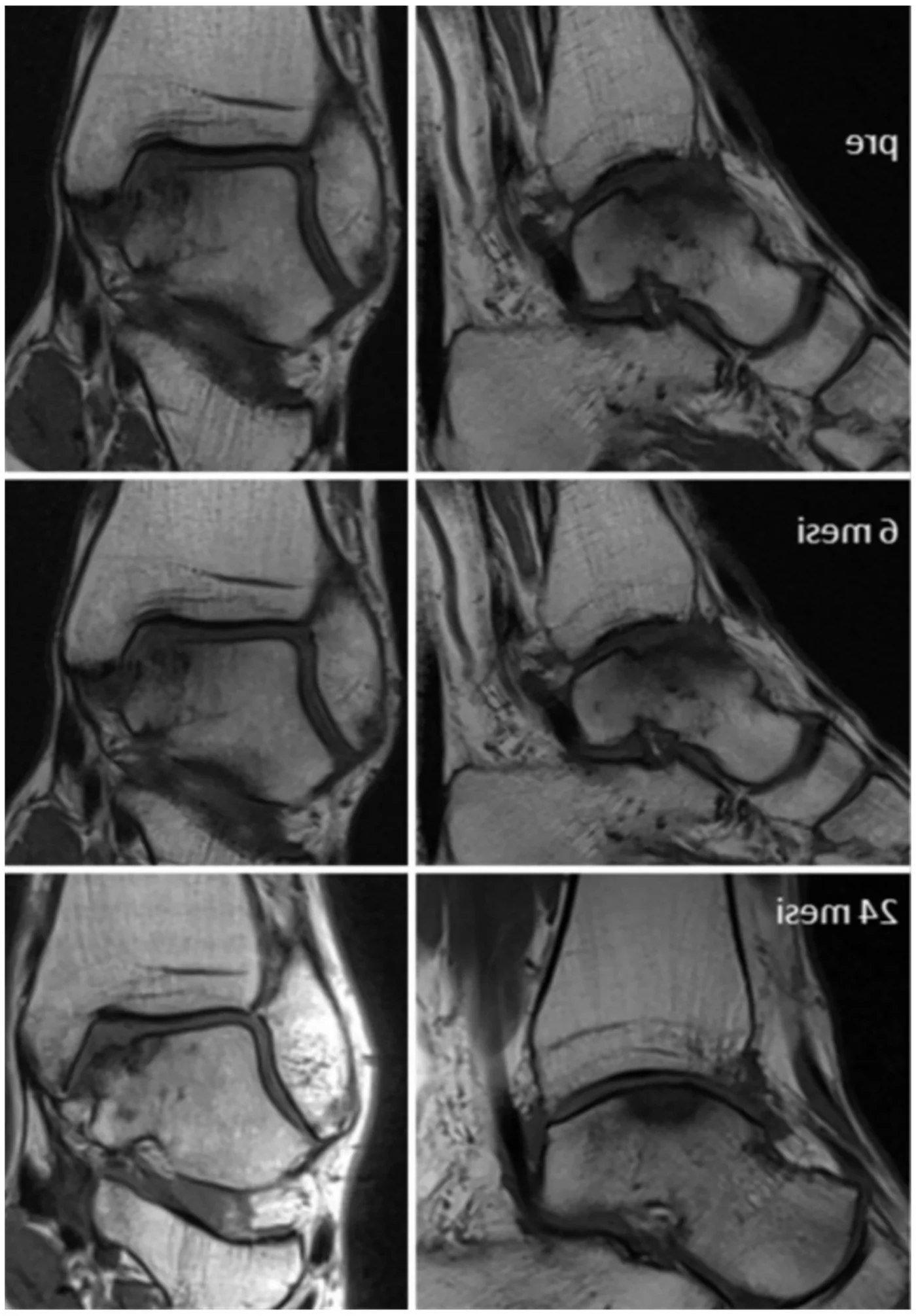
Representative serial MRI from one patient at the preoperative, 6-month, and 24-month time points. The intermediate image is illustrative and is not used as evidence of a complete cohort-level longitudinal dataset.

MOCART, Magnetic Resonance Observation of Cartilage Repair Tissue. These findings describe repair-tissue morphology and do not provide histological confirmation of normal hyaline-cartilage regeneration.

No intraoperative complications were recorded. Three of the 15 patients (20.0%) developed symptomatic anterior impingement and underwent arthroscopic reoperation at 7, 8, and 9 months, respectively. These procedures are reported as reoperations rather than routine second-look arthroscopies. The cartilage-like repair tissue observed during these procedures is described only as a finding in the three symptomatic patients and is not generalized to the remainder of the cohort.

## Discussion

The principal finding of this exploratory retrospective case series is that the 15 patients with complete 24-month follow-up after the combined single-stage procedure showed substantial improvement in pain and ankle-hindfoot functional scores together with generally favorable MRI morphology of repair tissue. The magnitude of the observed clinical change is consistent with previous reports of bone marrow-derived cell transplantation and matrix-assisted procedures for OLT (33, 42–48). These results should, however, be interpreted as outcomes after the complete combined procedure rather than as evidence for an independent effect of the bone marrow-derived cellular component.

The intervention included debridement, microfracture-based bone-marrow stimulation, application of autologous bone marrow-derived cell concentrate, a type I collagen scaffold, and fibrin-glue stabilization. Each component may contribute to the biological and mechanical environment of repair. In the absence of a comparator group, the present study cannot determine the relative contribution of these components or establish superiority over microfracture, matrix-augmented bone-marrow stimulation, osteochondral transplantation, or autologous chondrocyte implantation. Previous comparative studies and consensus statements show that treatment selection remains dependent on lesion characteristics and clinical context (45, 46, 49–54).

The terminology used for the cellular product is particularly important. The aspirate in this series was centrifuged intraoperatively and implanted without *ex vivo* expansion or phenotypic selection. It was therefore a heterogeneous bone marrow-derived cell concentrate rather than an isolated population of characterized MSCs. The revised manuscript adopts this terminology throughout the main text and reports the preparation according to the available DOSES elements (34). This distinction is consistent with evidence showing that the identification and quantification of MSCs in fresh bone marrow aspirate concentrate remain methodologically uncertain (35).

At 24 months, complete defect filling was observed in 80.0% of patients and complete integration with adjacent tissue in 93.3%, with a mean MOCART score of 83.7 ± 14.4. These values suggest favorable structural repair in most of the analyzed cohort and are broadly compatible with previous MRI reports after single-stage bone marrow-derived procedures (42–44, 46). Nevertheless, MOCART is a morphological score. It does not demonstrate that the repair tissue is histologically identical to native hyaline cartilage. For this reason, the revised manuscript avoids the term “cartilage regeneration” when interpreting the present MRI data and instead refers to repair tissue or cartilage-like repair tissue.

Persistent T2 hyperintensity was present in 80.0% of patients despite favorable clinical scores. This observation further illustrates that morphological or signal abnormalities on MRI do not necessarily parallel symptoms at a single time point. Because the submitted dataset did not support a fully documented correlation analysis, the previously reported MOCART-AOFAS correlation testing was removed rather than overinterpreted.

Three patients required arthroscopic reoperation for symptomatic anterior impingement. Their arthroscopic findings provide useful descriptive information but are subject to clear selection bias, because second-look visualization was performed only in symptomatic patients. These findings cannot therefore be extrapolated to the entire cohort or used as histological validation of the MRI appearance. Reporting these procedures as reoperations also provides a more transparent assessment of the postoperative course than describing the procedure broadly as “safe.”

Single-stage strategies have practical appeal because harvesting, processing, scaffold loading, and implantation can be completed during one operative episode, avoiding the culture and second implantation procedure required by conventional autologous chondrocyte implantation (47, 48, 55–65). This logistical feature is an inherent characteristic of the technique, but the present study was not designed to compare resource use, cost, length of stay, or patient burden with other procedures and therefore does not support claims of clinical or economic superiority.

### Limitation

Some limitations should be considered when interpreting these findings. The retrospective single-center design and the relatively small final cohort limit the generalizability of the results. In addition, 11 of the 26 screened patients did not have sufficiently complete clinical and/or imaging follow-up for inclusion in the 24-month analysis, which may have introduced some degree of selection bias. The available retrospective records did not allow a reliable comparison between included and excluded patients. The absence of a comparator group and the multimodal nature of the procedure also prevent the contribution of the bone marrow-derived cell concentrate from being assessed separately from the other components of treatment. Finally, MRI findings provide morphological information on repair tissue but cannot confirm its histological composition.

## Conclusion

In this retrospective cohort, the combined single-stage treatment of chronic osteochondral talar lesions was associated with improved 24-month pain and functional scores and favorable MRI morphology of repair tissue. These preliminary findings do not establish comparative efficacy, superiority over alternative procedures, or an independent effect of the bone marrow-derived cellular component. Larger prospective controlled studies using standardized cell-product characterization, complete longitudinal follow-up, and longer-term clinical and imaging outcomes are required.

## Data Availability

The original contributions presented in the study are included in the article/Supplementary Material, further inquiries can be directed to the corresponding author.

## References

[1] McGahan PJ Pinney SJ. Current concept review: osteochondral lesions of the talus. Foot Ankle Int. (2010) 31:90–101. 10.3113/FAI.2010.0090 20067729

[2] Hintermann B Boss A Schäfer D. Arthroscopic findings in patients with chronic ankle instability. Am J Sports Med. (2002) 30:402–9. 10.1177/03635465020300031601 12016082

[3] Saxena A Eakin C. Articular talar injuries in athletes: results of microfracture and autogenous bone graft. Am J Sports Med. (2007) 35:1680–7. 10.1177/0363546507303561 17656737

[4] Leontaritis N Hinojosa L Panchbhavi VK. Arthroscopically detected intra-articular lesions associated with acute ankle fractures. J Bone Joint Surg Am. (2009) 91:333–9. 10.2106/JBJS.H.00584 19181977

[5] Berndt AL Harty M. Transchondral fractures (osteochondritis dissecans) of the talus. J Bone Joint Surg Am. (1959) 41-A:988–1020. 10.2106/00004623-195941060-00002 13849029

[6] Flick AB Gould N. Osteochondritis dissecans of the talus (transchondral fractures of the talus): review of the literature and new surgical approach for medial dome lesions. Foot Ankle. (1985) 5:165–85. 10.1177/107110078500500403 3830846

[7] Kumai T Takakura Y Higashiyama I Tamai S. Arthroscopic drilling for the treatment of osteochondral lesions of the talus. J Bone Joint Surg Am. (1999) 81:1229–35. 10.2106/00004623-199909000-00004 10505519

[8] Prakash D Learmonth D. Natural progression of osteo-chondral defect in the femoral condyle. Knee. (2002) 9:7–10. 10.1016/s0968-0160(01)00133-8 11830374

[9] Tribe HC McEwan J Taylor H Oreffo ROC Tare RS. Mesenchymal stem cells: potential role in the treatment of osteochondral lesions of the ankle. Biotechnol J. (2017) 12:1700070. 10.1002/biot.201700070 29068173 PMC5765412

[10] DePalma AF McKeever CD Subin DK. Process of repair of articular cartilage demonstrated by histology and autoradiography with tritiated thymidine. Clin Orthop Relat Res. (1966) 48:229–42. 10.1097/00003086-196609000-00028 5334343

[11] Manunta AF Zedde P Cudoni S Caggiari G Pintus G. Early joint degeneration and antagonism between growth factors and reactive oxygen species. Is non-surgical management possible? Joints. (2015) 3:123–8. 10.11138/jts/2015.3.3.123 26889468 PMC4732778

[12] Kuettner KE Cole AA. Cartilage degeneration in different human joints. Osteoarthritis Cartilage. (2005) 13:93–103. 10.1016/j.joca.2004.11.006 15694570

[13] Ateshian GA Soslowsky LJ Mow VC. Quantitation of articular surface topography and cartilage thickness in knee joints using stereophotogrammetry. J Biomech. (1991) 24:761–76. 10.1016/0021-9290(91)90340-s 1918099

[14] Bhosale AM Richardson JB. Articular cartilage: structure, injuries and review of management. Br Med Bull. (2008) 87:77–95. 10.1093/bmb/ldn025 18676397

[15] Badekas T Takvorian M Souras N. Treatment principles for osteochondral lesions in foot and ankle. Int Orthop. (2013) 37:1697–706. 10.1007/s00264-013-2076-1 23982639 PMC3764304

[16] Verhagen RAW Struijs PAA Bossuyt PMM Van Dijk CN. Systematic review of treatment strategies for osteochondral defects of the talar dome. Foot Ankle Clin. (2003) 8:233–42, viii–ix. 10.1016/s1083-7515(02)00064-5 12911238

[17] Tol JL Struijs PA Bossuyt PM Verhagen RA Van Dijk CN. Treatment strategies in osteochondral defects of the talar dome: a systematic review. Foot Ankle Int. (2000) 21:119–26. 10.1177/107110070002100205 10694023

[18] Gobbi A Francisco RA Lubowitz JH Allegra F Canata G. Osteochondral lesions of the talus: randomized controlled trial comparing chondroplasty, microfracture, and osteochondral autograft transplantation. Arthroscopy. (2006) 22:1085–92. 10.1016/j.arthro.2006.05.016 17027406

[19] Rungprai C Tennant JN Gentry RD Phisitkul P. Management of osteochondral lesions of the talar dome. Open Orthop J. (2017) 11:743–61. 10.2174/1874325001711010743 28979588 PMC5620407

[20] O’Driscoll SW. The healing and regeneration of articular cartilage. J Bone Joint Surg Am. (1998) 80:1795–812. 10.2106/00004623-199812000-00011 9875939

[21] Giannini S Buda R Faldini C Vannini F Bevoni R Grandi G. Surgical treatment of osteochondral lesions of the talus in young active patients. J Bone Joint Surg Am. (2005) 87(Suppl 2):28–41. 10.2106/JBJS.E.00516 16326721

[22] Posadzy M Desimpel J Vanhoenacker F. Staging of osteochondral lesions of the talus: mRI and cone beam CT. J Belg Soc Radiol. (2017) 101:1. 10.5334/jbr-btr.1377 30498800 PMC6251071

[23] Ferkel RD Zanotti RM Komenda GA Sgaglione NA Cheng MS Applegate GR. Arthroscopic treatment of chronic osteochondral lesions of the talus: long-term results. Am J Sports Med. (2008) 36:1750–62. 10.1177/0363546508316773 18753679

[24] Cheng MS Ferkel RD Applegate GR. Osteochondral lesions of the talus: a radiologic and surgical comparison. In: 62nd Annual Meeting of the American Academy of Orthopaedic Surgeons; February 16 -21, 1995. Orlando, FL: American Academy of Orthopaedic Surgeons (1995). Oral presentation.

[25] Cuttica DJ Smith WB Hyer CF Philbin TM Berlet GC. Osteochondral lesions of the talus: predictors of clinical outcome. Foot Ankle Int. (2011) 32:1045–51. 10.3113/FAI.2011.1045 22338953

[26] Hannon CP Smyth NA Murawski CD Savage-Elliott BA Deyer TW Calder JDF. Osteochondral lesions of the talus: aspects of current management. Bone Joint J. (2014) 96-B:164–71. 10.1302/0301-620X.96B2.31637 24493179

[27] Chuckpaiwong B Berkson EM Theodore GH. Microfracture for osteochondral lesions of the ankle: outcome analysis and outcome predictors of 105 cases. Arthroscopy. (2008) 24:106–12. 10.1016/j.arthro.2007.07.022 18182210

[28] Beris AE Lykissas MG Papageorgiou CD Georgoulis AD. Advances in articular cartilage repair. Injury. (2005) 36(Suppl 4):S14–23. 10.1016/j.injury.2005.10.007 16291320

[29] Galois L Freyria A-M Herbage D Mainard D. [Cartilage tissue engineering: state- of-the-art and future approaches]. Pathol Biol (Paris). (2005) 53:590–8. 10.1016/j.patbio.2004.12.019 16364811

[30] Shive MS Stanish WD McCormack R Forriol F Mohtadi N Pelet S. BST- CarGel® treatment maintains cartilage repair superiority over microfracture at 5 years in a multicenter randomized controlled trial. Cartilage. (2015) 6:62–72. 10.1177/1947603514562064 26069709 PMC4462252

[31] Tare RS Kanczler J Aarvold A Jones AMH Dunlop DG Oreffo ROC. Skeletal stem cells and bone regeneration: translational strategies from bench to clinic. Proc Inst Mech Eng H. (2010) 224:1455–70. 10.1243/09544119JEIM750 21287831

[32] Gao F Chiu SM Motan DAL Zhang Z Chen L Ji H-L. Mesenchymal stem cells and immunomodulation: current status and future prospects. Cell Death Dis. (2016) 7:e2062. 10.1038/cddis.2015.327 26794657 PMC4816164

[33] Vannini F Cavallo M Ramponi L Castagnini F Massimi S Giannini S. Return to sports after bone marrow-derived cell transplantation for osteochondral lesions of the talus. Cartilage. (2017) 8:80–7. 10.1177/1947603516642574 27994723 PMC5154421

[34] Murray IR Chahla J Safran MR Krych AJ Saris DBF Caplan AI. International expert consensus on a cell therapy communication tool: dOSES. J Bone Joint Surg Am. (2019) 101:904–11. 10.2106/JBJS.18.00915 31094982 PMC7292498

[35] Ruoss S Walker JT Nasamran CA Fisch KM Paez CJ Parekh JN. Strategies to identify mesenchymal stromal cells in minimally manipulated human bone marrow aspirate concentrate lack consensus. Am J Sports Med. (2021) 49:1313–22. 10.1177/0363546521993788 33646886 PMC8409176

[36] Von Elm E Altman DG Egger M Pocock SJ Gøtzsche PC Vandenbroucke JP. The strengthening the reporting of observational studies in epidemiology (STROBE) statement: guidelines for reporting observational studies. J Clin Epidemiol. (2008) 61:344–9. 10.1016/j.jclinepi.2007.11.008 18313558

[37] Outerbridge RE. The etiology of chondromalacia patellae. J Bone Joint Surg Br. (1961) 43-B:752–7. 10.1302/0301-620X.43B4.752 14038135

[38] Giannini S Vannini F. Operative treatment of osteochondral lesions of the talar dome: current concepts review. Foot Ankle Int. (2004) 25:168–75. 10.1177/107110070402500311 15006340

[39] Kitaoka HB Alexander IJ Adelaar RS Nunley JA Myerson MS Sanders M. Clinical rating systems for the ankle-hindfoot, midfoot, hallux, and lesser toes. Foot Ankle Int. (1994) 15:349–53. 10.1177/107110079401500701 7951968

[40] Bijur PE Silver W Gallagher EJ. Reliability of the visual analog scale for measurement of acute pain. Acad Emerg Med. (2001) 8:1153–7. 10.1111/j.1553-2712.2001.tb01132.x 11733293

[41] Marlovits S Singer P Zeller P Mandl I Haller J Trattnig S. Magnetic resonance observation of cartilage repair tissue (MOCART) for the evaluation of autologous chondrocyte transplantation: determination of interobserver variability and correlation to clinical outcome after 2 years. Eur J Radiol. (2006) 57:16–23. 10.1016/j.ejrad.2005.08.007 16203119

[42] Giannini S Buda R Battaglia M Cavallo M Ruffilli A Ramponi L. One-step repair in talar osteochondral lesions: 4-year clinical results and t2-mapping capability in outcome prediction. Am J Sports Med. (2013) 41:511–8. 10.1177/0363546512467622 23221772

[43] Puddu L Altamore F Santandrea A Mercurio D Caggiari G Della Sala S. Surgical treatment of talar osteo-chondral lesions with micro-fractures, mesenchymal cells grafting on membrane, or allograft: mid-term clinical and magnetic resonance assessment. J Orthop. (2020) 21:416–20. 10.1016/j.jor.2020.08.012 32921950 PMC7474341

[44] Buda R Castagnini F Cavallo M Ramponi L Vannini F Giannini S. One-step” bone marrow-derived cells transplantation and joint debridement for osteochondral lesions of the talus in ankle osteoarthritis: clinical and radiological outcomes at 36 months. Arch Orthop Trauma Surg. (2016) 136:107–16. 10.1007/s00402-015-2344-1 26471987

[45] Richter M Zech S Meissner S Naef I. Comparison matrix-associated stem cell transplantation (MAST) with autologous matrix induced chondrogenesis plus peripheral blood concentrate (AMIC+PBC) in chondral lesions at the ankle-A clinical matched-patient analysis. Foot Ankle Surg. (2020) 26:669–75. 10.1016/j.fas.2019.08.009 31548148

[46] Kim BS Na Y Jang D-S. Outcomes of bone marrow aspirate concentrate and matrix-induced chondrogenesis (BMIC) for treatment of osteochondral lesions of the talus. Foot Ankle Surg. (2022) 28:944–9. 10.1016/j.fas.2022.01.005 35063361

[47] Giannini S Buda R Vannini F Cavallo M Grigolo B. One-step bone marrow- derived cell transplantation in talar osteochondral lesions. Clin Orthop Relat Res. (2009) 467:3307–20. 10.1007/s11999-009-0885-8 19449082 PMC2772930

[48] Giannini S Buda R Cavallo M Ruffilli A Cenacchi A Cavallo C. Cartilage repair evolution in post-traumatic osteochondral lesions of the talus: from open field autologous chondrocyte to bone-marrow-derived cells transplantation. Injury. (2010) 41:1196–203. 10.1016/j.injury.2010.09.028 20934692

[49] Choi WJ Park KK Kim BS Lee JW. Osteochondral lesion of the talus: is there a critical defect size for poor outcome? Am J Sports Med. (2009) 37:1974–80. 10.1177/0363546509335765 19654429

[50] Ramponi L Yasui Y Murawski CD Ferkel RD DiGiovanni CW Kerkhoffs GMMJ. Lesion size is a predictor of clinical outcomes after bone marrow stimulation for osteochondral lesions of the talus: a systematic review. Am J Sports Med. (2017) 45:1698–705. 10.1177/0363546516668292 27852595

[51] Becher C Malahias MA Ali MM Maffulli N Thermann H. Arthroscopic microfracture vs. Arthroscopic autologous matrix-induced chondrogenesis for the treatment of articular cartilage defects of the talus. Knee Surg Sports Traumatol Arthrosc. (2019) 27:2731–6. 10.1007/s00167-018-5278-7 30392029

[52] Rothrauff BB Murawski CD Angthong C Becher C Nehrer S Niemeyer P. Scaffold-based therapies: proceedings of the international consensus meeting on cartilage repair of the ankle. Foot Ankle Int. (2018) 39:41S–7. 10.1177/1071100718781864 30215312

[53] Loveday D Clifton R Robinson A. Interventions for treating osteochondral defects of the talus in adults. Cochrane Database Syst Rev. (2010) 2010(8):CD008104. 10.1002/14651858.CD008104.pub2 20687094 PMC13310591

[54] Murawski CD Kennedy JG. Operative treatment of osteochondral lesions of the talus. J Bone Joint Surg Am. (2013) 95:1045–54. 10.2106/JBJS.L.00773 23780543

[55] Kon E Gobbi A Filardo G Delcogliano M Zaffagnini S Marcacci M. Arthroscopic second-generation autologous chondrocyte implantation compared with microfracture for chondral lesions of the knee: prospective nonrandomized study at 5 years. Am J Sports Med. (2009) 37:33–41. 10.1177/0363546508323256 19059899

[56] Baums MH Heidrich G Schultz W Steckel H Kahl E Klinger H-M. Autologous chondrocyte transplantation for treating cartilage defects of the talus. J Bone Joint Surg Am. (2006) 88:303–8. 10.2106/JBJS.E.00033 16452741

[57] Puddu L Lugani G Perusi F Brunialti D Cont F Ciatti C. Acute treatment of osteochondral detachment following patellar dislocation: clinical and short-term MRI follow-up. Life (Basel). (2024) 14(1):85. 10.3390/life14010085 38255700 PMC10820480

[58] Manunta AF Zedde P Cudoni S Caggiari G Pintus G. Early joint degeneration and antagonism between growth factors and reactive oxygen species. Is non-surgical management possible? Joints. (2016) 3(3):123–8. 10.11138/jts/2015.3.3.123 26889468 PMC4732778

[59] Wakitani S Nawata M Tensho K Okabe T Machida H Ohgushi H. Repair of articular cartilage defects in the patello-femoral joint with autologous bone marrow mesenchymal cell transplantation: three case reports involving nine defects in five knees. J Tissue Eng Regen Med. (2007) 1:74–9. 10.1002/term.8 18038395

[60] Braile A De Cicco A Liguori S Matteo VD Conza G Vasso M. Preliminary report on the time-related effect of a single autologous adipose-derived mesenchymal stem cells injection in hip osteoarthritis: a retrospective observational study. Adv Orthop. (2025) 2025:3424035. 10.1155/aort/3424035 40552343 PMC12185193

[61] Conti MS Ellington JK Behrens SB. Osteochondral defects of the talus: how to treat without an osteotomy. Clin Sports Med. (2020) 39:893–909. 10.1016/j.csm.2020.07.002 32892974

[62] Pittenger MF Mackay AM Beck SC Jaiswal RK Douglas R Mosca JD. Multilineage potential of adult human mesenchymal stem cells. Science. (1999) 284:143–7. 10.1126/science.284.5411.143 10102814

[63] Sakaguchi Y Sekiya I Yagishita K Muneta T. Comparison of human stem cells derived from various mesenchymal tissues: superiority of synovium as a cell source. Arthritis Rheum. (2005) 52:2521–9. 10.1002/art.21212 16052568

[64] Saw K-Y Anz A Siew-Yoke Jee C Merican S Ching-Soong Ng R Roohi SA. Articular cartilage regeneration with autologous peripheral blood stem cells versus hyaluronic acid: a randomized controlled trial. Arthroscopy. (2013) 29:684–94. 10.1016/j.arthro.2012.12.008 23380230

[65] Selmi TS Verdonk P Chambat P Dubrana F Potel J-F Barnouin L. Autologous chondrocyte implantation in a novel alginate-agarose hydrogel: outcome at two years. J Bone Joint Surg Br. (2008) 90:597–604. doi: 10.1302/0301- 620X.90B5.20360 18450625 10.1302/0301-620X.90B5.20360

