# Supplementary material for: Single-stage matrix-assisted bone marrow-derived cell concentrate implantation for osteochondral talar lesions: a 24-month clinical and MRI follow-up
Source: Front Surg. 2026 Oct 30;13:1941433. doi: 10.3389/fsurg.2026.1941433 (PMC13759736; doi:10.3389/fsurg.2026.1941433)
Supplement: Supplementary file 1 [file Table1.docx]

Table 1. Demographic and lesion characteristics of all patients treated with autologous BM-DCT from January 2016 to December 2020 (n = 26).

|  |  | **Mean (SD)** |
| --- | --- | --- |
| Age (years) |  | 34.0 (9.5) |
| BMI |  | 25.0 (2.5) |
| Follow-up (months) |  | 38.0 (3.2) |
| Time from diagnosis to surgery (months) |  | 16.0 (12.4) |
| Lesion area (cm²) |  | 1.7 (2.1) |
| Lesion depth (mm) |  | 6.0 (1.2) |
|  |  | n (%) |
| Sex | Male | 16 (61.5%) |
|  | Female | 10 (38.5%) |
| Lesion location | Medial | 18 (69.2%) |
|  | Lateral | 8 (30.8%) |
| Lesion side | Left | 12 (46.2%) |
|  | Right | 14 (53.8%) |
| Traumatic event | Positive | 20 (76.9%) |
|  | Negative | 6 (23.1%) |

BMI = body mass index; BM-DCT = bone marrow-derived cell transplantation; SD = standard deviation.
