# Supplementary material for: Single-stage matrix-assisted bone marrow-derived cell concentrate implantation for osteochondral talar lesions: a 24-month clinical and MRI follow-up
Source: Front Surg. 2026 Oct 30;13:1941433. doi: 10.3389/fsurg.2026.1941433 (PMC13759736; doi:10.3389/fsurg.2026.1941433)
Supplement: Supplementary file 2 [file Table2.docx]

Table 2. AOFAS and VAS scores before surgery and at 24-month follow-up in the final study cohort (n = 15).

|  | **Pre-surgery, mean (SD)** | **24-month follow-up, mean (SD)** |
| --- | --- | --- |
| AOFAS | 57.3 (12.0) | 92.4 (7.1) |
| VAS | 4.7 (1.2) | 1.8 (0.8) |

AOFAS = American Orthopaedic Foot & Ankle Society score; SD = standard deviation; VAS = Visual Analog Scale.
Note: VAS SD values were corrected from 12.0 and 8.0 to 1.2 and 0.8, respectively, assuming decimal-point transcription errors.
