# Supplementary material for: Single-stage matrix-assisted bone marrow-derived cell concentrate implantation for osteochondral talar lesions: a 24-month clinical and MRI follow-up
Source: Front Surg. 2026 Oct 30;13:1941433. doi: 10.3389/fsurg.2026.1941433 (PMC13759736; doi:10.3389/fsurg.2026.1941433)
Supplement: Supplementary file 3 [file Table3.docx]

Table 3. MRI evaluation of repair tissue in patients with at least 24 months of follow-up (n = 15).

| **Parameter** | **Grade** | **n (%)** |
| --- | --- | --- |
| Filling of the osteochondral defect | Complete | 12 (80.0%) |
|  | Hypertrophic | 2 (13.3%) |
|  | Incomplete >50% | 1 (6.7%) |
|  | Incomplete <50% | 0 (0.0%) |
|  | Exposure of the subchondral bone | 0 (0.0%) |
| Scaffold edge integration | Complete | 14 (93.3%) |
|  | Incomplete >50% | 0 (0.0%) |
|  | Incomplete <50% | 1 (6.7%) |
|  | Incomplete with demarcated edges | 0 (0.0%) |
| Scaffold surface | Preserved | 12 (80.0%) |
|  | Damaged <50% compared with the surrounding cartilage | 3 (20.0%) |
|  | Damaged >50% compared with the surrounding cartilage | 0 (0.0%) |
| Scaffold structure | Homogeneous | 12 (80.0%) |
|  | Non-homogeneous | 3 (20.0%) |
| Scaffold signal intensity on FSE | Isointense | 12 (80.0%) |
|  | Moderately hyperintense | 3 (20.0%) |
|  | Markedly hyperintense | 0 (0.0%) |
| Integrity of the subchondral lamina | Preserved | 14 (93.3%) |
|  | Not preserved | 1 (6.7%) |
| Integrity of the subchondral bone | Preserved | 0 (0.0%) |
|  | Not preserved | 15 (100.0%) |
| Presence of adhesions | Positive | 0 (0.0%) |
|  | Negative | 15 (100.0%) |
| Presence of joint effusion | Positive | 5 (33.3%) |
|  | Negative | 10 (66.7%) |
| Signal intensity on T2-weighted FSE sequence | Isointense | 3 (20.0%) |
|  | Moderately hyperintense | 12 (80.0%) |
|  | Markedly hyperintense | 0 (0.0%) |

FSE = fast spin echo; MRI = magnetic resonance imaging.
Note: percentages were recalculated using n = 15. The moderately hyperintense category for scaffold signal intensity on FSE was corrected to 3 (20.0%) so that the category totals sum to n = 15.
