# Supplementary material for: Single-stage matrix-assisted bone marrow-derived cell concentrate implantation for osteochondral talar lesions: a 24-month clinical and MRI follow-up
Source: Front Surg. 2026 Oct 30;13:1941433. doi: 10.3389/fsurg.2026.1941433 (PMC13759736; doi:10.3389/fsurg.2026.1941433)
Supplement: Supplementary file 4 [file Table4.docx]

Table 4. Mean MOCART score subcategories calculated by the four surgeons.

| **Category** | **Mean MOCART score** | **SD** | **Interrater agreement** | |
| --- | --- | --- | --- | --- |
|  |  |  | **Krippendorff's alpha** | **Average Cohen's kappa** |
| Defect fill | 18.25 | 3.30 | 0.809 | 0.816 |
| Cartilage interface | 13.50 | 3.94 | 0.848 | 0.848 |
| Surface | 8.92 | 2.08 | 0.902 | 0.907 |
| Adhesion | 4.25 | 1.80 | 0.870 | 0.881 |
| Structure | 4.08 | 1.95 | 0.899 | 0.881 |
| Signal intensity | 26.00 | 8.07 | 0.793 | 0.792 |
| Subchondral lamina | 4.08 | 1.95 | 0.889 | 0.881 |
| Subchondral bone | 0.58 | 1.62 | 0.839 | 0.817 |
| Effusion | 3.50 | 2.31 | 0.842 | 0.845 |

MOCART = Magnetic Resonance Observation of Cartilage Repair Tissue; SD = standard deviation.
