# Supplementary material for: Single-stage matrix-assisted bone marrow-derived cell concentrate implantation for osteochondral talar lesions: a 24-month clinical and MRI follow-up
Source: Front Surg. 2026 Oct 30;13:1941433. doi: 10.3389/fsurg.2026.1941433 (PMC13759736; doi:10.3389/fsurg.2026.1941433)
Supplement: Supplementary file 5 [file Table5.docx]

Table 5. Data from patients who underwent arthroscopic second-look evaluation (n = 3).

| **Patient** | **Time of arthroscopic second-look evaluation (months)** | **AOFAS** | | **Ferkel and Cheng classification** |
| --- | --- | --- | --- | --- |
|  |  | **Pre-surgery** | **12-month follow-up** |  |
| Patient 1 | 7 | 42 | 87 | A |
| Patient 2 | 8 | 45 | 84 | A |
| Patient 3 | 9 | 38 | 92 | C |
|  |  |  |  |  |
| AOFAS mean |  | 41.7 | 87.7 |  |
| AOFAS SD |  | 3.5 | 4.1 |  |

AOFAS = American Orthopaedic Foot & Ankle Society score; SD = standard deviation.
